# Comparison of 3Mixtatin and Mineral Trioxide Aggregate in Primary Teeth Pulpotomy: A Systematic Review

**DOI:** 10.7759/cureus.77284

**Published:** 2025-01-11

**Authors:** V Lalitha Priya, Kavitha Ramar

**Affiliations:** 1 Department of Pediatric and Preventive Dentistry, SRM Kattankulathur Dental College and Hospital, Chennai, IND

**Keywords:** 3mixtatin, calcium silicate materials, mineral trioxide aggregate, pediatric dentistry, primary teeth pulpotomy

## Abstract

The aim of this systematic review was to evaluate the clinical and radiographic success rates of primary teeth pulpotomy using 3Mixtatin compared to mineral trioxide aggregate (MTA). A comprehensive literature search identified relevant studies, which were screened for inclusion, with the risk of bias assessed using Cochrane collaboration tools. Data on success rates, follow-up periods, and methodological quality were extracted, and the protocol was registered in PROSPERO (ID: CRD42024562155). Among the four analyzed studies, Jamali et al. reported 24-month clinical success rates of 90.5%, 88.1%, and 78.9% for 3Mixtatin, MTA, and formocresol, respectively. Chak et al. observed a 95.7% clinical success rate for 3Mixtatin over 12 months, with no data on MTA, while Mushtaq et al. found comparable success rates for both 3Mixtatin and MTA. Reddy et al. reported clinical and radiographic success rates of 91.7% and 81.7% for formocresol, 100% and 91.7% for MTA, and 95% and 85% for 3Mixtatin at 6 and 12 months, respectively. Radiographically, 3Mixtatin demonstrated slightly better short-term outcomes than MTA. These findings suggest that both 3Mixtatin and MTA are effective for pulpotomy in primary dentition, with 3Mixtatin offering potential advantages due to its antibiotic and statin properties that may enhance healing and antibacterial effects. Additional studies involving larger and more diverse populations are required to confirm these findings.

## Introduction and background

Pulpotomy is a vital procedure in pediatric dentistry that focuses on maintaining the health and functionality of primary teeth impacted by caries or injury [1]. The primary goal of pulpotomy is to preserve the health and function of the remnant pulp tissues, even after the removal of the infected or inflamed coronal portion [1,2]. This vital pulp procedure has gained popularity as a viable alternative to pulpectomy because of its benefits, including shorter chair time, lower cost, better preservation of the structure of the tooth, enhanced regenerative capacity of the remaining pulp and enhanced extended clinical outcomes and radiographic results [1,3,4]. Material optimal for pulpotomy should possess effective sealing properties, biocompatibility, bio-inductive, as well as the capability to eradicate bacteria while preserving as well as promoting the healing of healthy radicular pulp [5-8]. The effectiveness of pulpotomy largely relies on the choice of medicament used to treat the remaining pulp and promote healing. Over the years, various materials have been introduced, with differing properties and outcomes. The options include materials and techniques such as formocresol, glutaraldehyde, paraformaldehyde, zinc oxide eugenol (ZOE), KRI paste, Ledermix, electrosurgery, ferric sulfate, mineral trioxide aggregate (MTA), specific growth factors, and lasers.

Among these, MTA has garnered significant attention in the field of dentistry and it has been lauded for its superior sealing ability, biocompatibility, bioactivity, hydrophilicity and its ability to promote the regeneration of dentin. Its use in pulpotomy has been associated with high clinical success rates, making it a preferred choice among dental practitioners. Despite its benefits, MTA has certain limitations such as difficult handling, prolonged setting time, susceptibility to discolouration and high cost [9,10].

However, the search for even better materials continues, leading to the development of innovative alternatives such as 3Mixtatin. It is a concoction material that combines simvastatin with triple antibiotic paste (TAP), which includes metronidazole, minocycline and ciprofloxacin [11]. It is also utilized for lesion sterilization and tissue repair (LSTR), and it is prognosticated for treating infected periapical lesions and instances of physiologic root resorption [12,13]. Also, it has recently been applied in primary teeth as a vital pulp therapy agent and in root canal procedures, owing to its crucial role in preserving the developmental and functional integrity of these teeth [14].

Despite the promising attributes of both MTA and 3Mixtatin, comparative studies evaluating their efficacy in pulpotomy are sparse. Considering the numerous publications on endodontics materials for primary teeth, the methodological studies, and the absence of systematic review comparing the performance of 3Mixtatin to MTA in pulpotomy procedures, this study is highly significant due to its relevance to the practice in clinics. The purpose of this study was to systematically review clinical trials to evaluate the clinical and radiographic success rates of primary teeth pulpotomy using 3Mixtatin compared to MTA.

## Review

Methods

Search Strategy

The following databases were systematically searched: PubMed, Scopus, Medline, Lilacs, Cochrane, Clinical Key, Embase, Wiley Library, and Google Scholar. This systematic review followed the guidelines of the Preferred Reporting Items for Systematic Reviews and Meta-Analyses (PRISMA) checklist, an evidence-based framework designed for comprehensive reporting in systematic reviews and meta-analyses. Under the protocol number CRD42024562155, the study protocol was entered into the International Prospective Register of Systematic Reviews (PROSPERO). This search was performed on May 15, 2024, and updated on December 29, 2024. Additionally, the reference sections of the included manuscripts were hand-searched.

Search Terms

Both controlled (Medical Subject Headings, MeSH) and uncontrolled (text words and their synonyms) vocabulary were used in the search method. Boolean operators (AND, OR, and NOT) were used to combine the search phrases, and each database was customized. For the present systematic review, a research question was formulated according to the PICOS framework (Population, Intervention, Comparison, Outcome, and Study Design): In deciduous teeth (P), does 3 Mixtatin pulpotomy (I), compared to mineral trioxide aggregate pulpotomy (C), result in superior clinical and radiographic success rates (O) as reported in randomized clinical trials (S)?. The initial search strategy employed in PubMed included: ((mineral trioxide aggregate) OR Mineral trioxide aggregate) OR Portland cement) OR tricalcium silicate) AND 3 Mixtatin) AND pulpotomy) AND primary teeth) OR deciduous teeth) OR primary dentition) OR deciduous dentition) NOT pulpectomy) NOT dental caries) NOT dental defects) NOT orthodontic treatments) NOT crowns) NOT permanent teeth) NOT injuries) NOT therapy) NOT pulp treatments) NOT occlusion) NOT malocclusion) NOT pulp) NOT Biodentine) NOT oral health. This strategy was adapted for searches in other databases.

Inclusion and Exclusion Criteria

The inclusion criteria were the clinical studies that provide data on both clinical and radiographic success rates, with a required minimum follow-up period of six months. Additionally, studies were eligible if they focused on the pediatric population and were published in English between 2000 and 2024. The exclusion criteria were case reports, review articles, conference abstracts or experts' opinions, in vitro studies, and studies that did not assess the two materials being evaluated.

Quality Assessment

A controlled clinical trial was identified, and the Cochrane methodology for assessing the quality of randomized controlled trials (RCTs) was applied in this systematic review of interventions for pulpotomy in primary teeth [15]. Each study was assessed for possible biases related to selection, performance, detection, attrition, and reporting.

Data Extraction and Synthesis

Reviewers separately extracted data from the included papers using a standardized form that was integrated into an electronic spreadsheet. This form captured all essential variables required for interpreting, calculating, and applying the results. Each study's clinical and radiographic success rates were assessed. All references have been organized with EndNote® X7 (Thomson Reuters, Philadelphia, PA) to manage citations and eliminate duplicate studies.

The bibliographic search retrieved 182 articles from electronic databases, which were deemed relevant and complementary, with tailored strategies applied for each database. The total number of citations retrieved from all databases was recorded to create a flowchart for article selection. This process tracked all stages, from the initial count of citations identified through the search strategies to the exclusion of duplicates, culminating in the final number of articles included.

Up until June 2024, the titles and abstracts of the identified studies were separately evaluated by two reviewers (LP and KR). Both the reviewers had received prior training and calibration for article selection based on the inclusion and exclusion criteria derived from the PICOS question. Any disagreements between the reviewers were addressed through discussion.

Results

Study Selection

**Figure 1:**
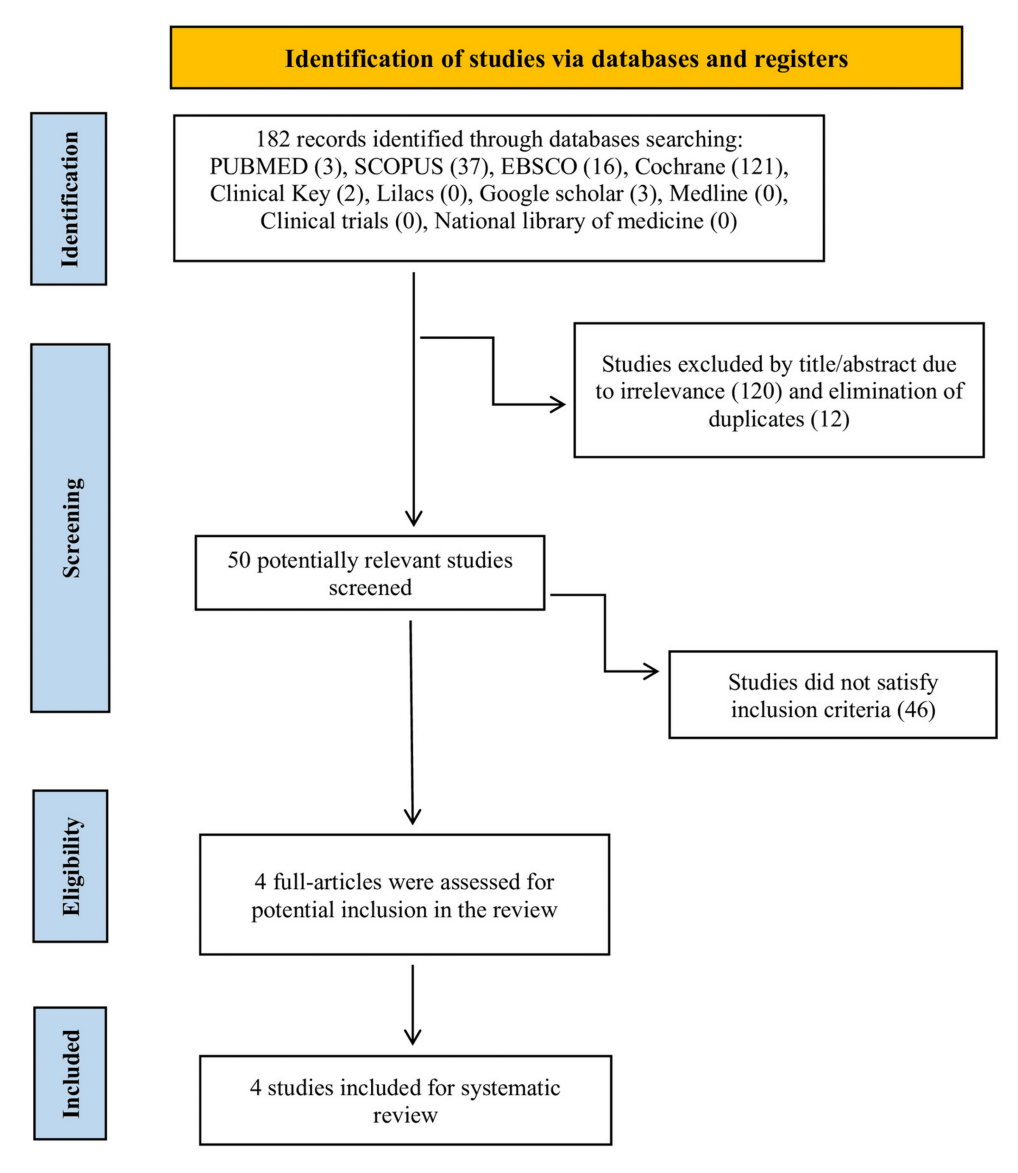
PRISMA flow diagram of the literature research process and results PRISMA: Preferred Reporting Items for Systematic Reviews and Meta-Analyses

EndNote® X7 was used to review all 182 articles that were obtained from the electronic databases. This helped to streamline the references and increase productivity. Initially, 120 studies were excluded based on their titles due to irrelevance and 12 duplicate articles were removed, leaving 50 articles for further review. During the eligibility assessment, articles were assigned to reviewers for independent screening of titles and abstracts, followed by consensus meetings at each stage. As a result, 178 studies that were not directly related to the research objectives were excluded. The remaining articles, shown in Figure 1, were considered potentially eligible and were then evaluated based on the predefined inclusion and exclusion criteria.

The studies selected in this phase were verified through a full-text review conducted independently by two reviewers, and inter-reviewer acceptance reliability was assessed throughout the process. Discrepancies between the reviewers were resolved through discussion. The agreement between the two reviewers was evaluated qualitatively, ensuring consistency in the study inclusion, data extraction, and quality assessment process. Ultimately, the full-text review confirmed the eligibility of four clinical studies.

Study Characteristics and Success Rate

Together with the clinical and radiographic success rates recorded at 12 and 24 months, Table 1 lists the descriptive characteristics of the studies that were part of this systematic review.

**Table 1:** Summary of reported key characteristics of four included studies in the systematic review FC: Formocresol; MTA: Mineral trioxide aggregate

Study Aspect	Jamali et al. [16]	Chak et al. [17]	Mushtaq et al. [18]	Reddy et al. [19]
Objective	Compare 3Mixtatin with other pulpotomy agents	Evaluate 3Mixtatin vs. MTA in pulpotomy	Assess clinical and radiographic outcomes of 3Mixtatin and MTA	Compare the clinical and radiographic success of 3Mixtatin with MTA and formocresol in pulpotomy
Duration	24 months	12 months	12 months	12 months
Sample Size	64 deciduous molars	64 deciduous molars	50 deciduous molars	60 deciduous molars
Group Details	3Mixtatin, FC, MTA	3Mixtatin, MTA	3Mixtatin, MTA	3Mixtatin, MTA, FC
Clinical Success Rate (%)	3Mixtatin: 90.5%, MTA: 88.1%, FC: 78.9%	3Mixtatin: 95.7%, MTA: Not specified	3Mixtatin: 95.7%; MTA: 96-100%	3Mixtatin: 86.6%; MTA: 93.4%; FC: 83.4%
Radiographic Success Rate (%)	3Mixtatin: Not specified, MTA: 88.1%, FC: Not specified	3Mixtatin: 91.3%, MTA: 75%	3Mixtatin: 91.3%, MTA: 96-100%	3Mixtatin: 83.4% MTA: 90% FC: 80%
Follow-Up Periods	6, 12, 18, and 24 months	3, 6, 9, and 12 months	3, 6, 9, and 12 months	6 and 12 months
Main Findings	3Mixtatin shows high success due to antibacterial and bio-inductive properties	3Mixtatin had a better outcome in radiographic success over MTA	Both materials showed high clinical success, no significant differences between groups	MTA showed better results. 3mixtatin can be considered as additional pulpotomy medicament.

The studies consistently report high success rates for 3Mixtatin, highlighting its potential as an effective alternative to traditional pulpotomy agents such as MTA and formocresol. Table 2 outlines the primary and secondary outcomes (clinical and radiographic criteria) of the four studies included in this review. Treatment failure in all four studies was determined by the presence of specific clinical and radiographic signs and symptoms. Based on these assessments, MTA demonstrated consistently high clinical and radiographic success rates, often comparable to 3Mixtatin. However, some studies highlighted that 3Mixtatin showed slightly better radiographic outcomes over shorter follow-up periods. Both 3Mixtatin and MTA have proven effective for pulpotomy in primary teeth, with evidence suggesting that 3Mixtatin may offer additional advantages due to its unique combination of antibiotic and statin properties, which enhance healing and antibacterial efficacy.

**Table 2:** Primary and secondary outcomes of the included studies FC: Formocresol; MTA: Mineral trioxide aggregate; PDL: Periodontal ligament space; IRR: Internal root resorption; ERR: External root resorption; FR: Furcal radiolucency; IBD: Inter-radicular bone destruction; PBD: Periapical bone destruction

Study	Primary Outcomes	Secondary Outcomes
Jamali et al. [16]	Success rate of pulpotomy (clinical and radiographic success) using 3Mixtatin compared to MTA and FC after a 24-month follow-up.	Sinus tract, tenderness to palpation and percussion, spontaneous pain or pain of long duration, swelling, presence of ERR or IRR, inter-radicular radiolucency, and periapical lesion.
Chak et al. [17]	Evaluates the efficacy of 3Mixtatin, a combination of simvastatin and 3Mix antibiotics, in comparison with MTA for pulpotomy in primary molars.	Absence of radiographic evidence of pulp degeneration, PDL widening, presence of IRR and ERR, FR/IBD, PBD.
Mushtaq et al. [18]	Evaluates the clinical and radiographic outcomes using 3Mixtatin as a pulpotomy medicament in primary molars with deep carious lesions	Clinical criteria: Presence of any postoperative pain, sensitivity to palpation and percussion, mobility along with examination of surrounding periodontium for the presence of any gingival inflammation, sinus tract or fistula. Radiographic criteria: Presence of ERR or IRR, PDL widening, furcation or periapical involvement.
Reddy et al. [19]	Evaluates the clinical and radiographic outcomes of 3Mixtatin compared to MTA and FC as pulpotomy agents in primary molars.	Presence of pain, tenderness to palpation and percussion, sinus tract, swelling, presence of ERR or IRR, inter-radicular radiolucency, periapical lesion.

Assessment of Risk of Bias

A study design-specific risk of bias instrument developed by the Cochrane Collaboration's risk of bias tool, also known as RoB 2 was used by two independent reviewers (LP, KR) to assess the risk of bias in the included studies [15]. This tool covers six areas related to random sequence generation, allocation concealment, blinding of participants and personnel, blind of outcomes assessment, incomplete outcome data and selective reporting. A comprehensive overview of the risk of bias information for the studies that were included in the analysis is given in Table 3.

**Table 3:** Summary of the risk of bias information for the included studies Article 1: Jamali et al. [16] Article 2: Chak et al. [17] Article 3: Mushtaq et al. [18] Article 4: Reddy et al. [19]

Aspect of Risk	Article 1	Article 2	Article 3	Article 4
Random Sequence Generation	Randomized; sequence generated using computer-generated random codes	Randomized with two coloured balls determining intervention	Randomized; sequence generated using computer-generated random codes​	Randomized; Not explicitly mentioned
Allocation Concealment	Not explicitly mentioned​	Not explicitly mentioned	Not explicitly mentioned	Not explicitly mentioned
Blinding of Participants and Personnel	Not explicitly mentioned	Blinding not explicitly mentioned, intervention may have been visible to participants	Not explicitly mentioned	Blinding not explicitly mentioned, intervention may have been visible to participants
Blinding of Outcome Assessment	Not explicitly mentioned	Blinding not explicitly mentioned	Not explicitly mentioned	Blinding not explicitly mentioned
Incomplete Outcome Data	Follow-up completed, six teeth excluded from the final analysis due to not meeting criteria	Not explicitly mentioned, but follow-up loss compensated by increasing the sample size	Follow-up completed, no exclusions reported	Follow-up completed, no exclusions reported
Selective Reporting	Results reported as planned	Results reported as planned	Results reported as planned	Results reported as planned
Other Bias	Ethical approval obtained, but study design details limited	Ethical approval mentioned; study design aligned with CONSORT guidelines	Ethical approval obtained, but study design details limited.	Ethical approval obtained, but study design details limited.

The risk of bias assessment revealed that random sequence generation was adequately described in Articles 1 and 3 using computer-generated codes, while Article 2 used coloured balls, and Article 4 did not specify the method. Allocation concealment and blinding (of participants, personnel, and outcome assessors) were not explicitly mentioned in any of the studies, with interventions possibly visible to participants in Articles 2 and 4. Incomplete outcome data were addressed in Articles 1, 3, and 4, but Article 2 managed follow-up loss by increasing the sample size. All studies reported outcomes as planned. Ethical approval was obtained across all studies, though design details were limited except in Article 2, which adhered to CONSORT guidelines.

Table 4 assesses the risk levels across the four articles, categorizing them as low, medium, or high. Random sequence generation was rated low for Articles 1, 2, and 3, but high for Article 4. Allocation concealment, participant and personnel blinding, and outcome assessment blinding were consistently rated as high risk across all articles, indicating potential selection, performance, and detection biases. Incomplete outcome data posed a medium risk for Articles 1 and 2, while Articles 3 and 4 had a low risk in this aspect. All studies reported outcomes as planned, indicating a low risk of selective reporting bias. Other biases, such as ethical approval and study design, were rated medium for Articles 1, 3, and 4, but low for Article 2.

**Table 4:** Interpretation of the risk levels as high, low, or medium for the four articles Article 1: Jamali et al. [16] Article 2: Chak et al. [17] Article 3: Mushtaq et al. [18] Article 4: Reddy et al. [19]

Aspect of Risk	Article 1	Article 2	Article 3	Article 4
Random Sequence Generation	Low	Low	Low	High
Allocation Concealment	High	High	High	High
Blinding of Participants and Personnel	High	High	High	High
Blinding of Outcome Assessment	High	High	High	High
Incomplete Outcome Data	Medium	Medium	Low	Low
Selective Reporting	Low	Low	Low	Low
Other Bias	Medium	Low	Medium	Medium

Level of Evidence

Table 5 presents the level of evidence for the four studies included in the systematic review, while Table 6 offers detailed information on the level of evidence and specific details for each study. The evidence levels ranged from Level II for the RCTs (Studies 1, 2 and 4) to Level III for the quasi-experimental study (Study 3). These levels correspond to the study designs and methodologies used, with RCTs generally offering higher-quality evidence due to their use of randomization and controlled conditions.

**Table 5:** Level of evidence in all four included studies in systematic review Study 1: Jamali et al. [16] Study 2: Chak et al. [17] Study 3: Mushtaq et al. [18] Study 4: Reddy et al. [19] MTA: Mineral trioxide aggregate; RCT: Randomized controlled trial

Study	Intervention	Comparison	Level of Evidence	Study Type
Study 1	3Mixtatin	MTA, formocresol	Level II	RCT
Study 2	3Mixtatin	MTA	Level II	RCT
Study 3	3Mixtatin	MTA	Level III	Quasi-experimental
Study 4	3Mixtatin	MTA, formocresol	Level II	RCT

**Table 6:** Level of evidence and details of four studies included in this systematic review Study 1: Jamali et al. [16] Study 2: Chak et al. [17] Study 3: Mushtaq et al. [18] Study 4: Reddy et al. [19] MTA: Mineral trioxide aggregate

Study	Study Design	Level of Evidence	Details
Study 1	Randomized Controlled Trial	Level II	- Participants: 114 children, 150 primary molars requiring pulpotomy. - Interventions: Compared MTA, Formocresol, and 3Mixtatin. - Methodology: Randomization done using RandList software, with allocation concealment.
Study 2	Randomized Controlled Trial	Level II	- Participants: Specific sample size not detailed in extract. - Interventions: Focused on MTA vs. 3Mixtatin vs. Formocresol. - Outcomes: Success rates and safety profiles evaluated over a set period.
Study 3	Comparative Clinical Study	Level III	- Participants: Not specified in extract, assumed to be children with molars needing treatment. - Interventions: 3Mixtatin compared to MTA. - Outcomes: Clinical success rates noted, with emphasis on non-randomized design.
Study 4	Comparative Clinical Study	Level II	- Participants: 100 children, 60 primary molars requiring pulpotomy. - Interventions: Compared MTA, Formocresol, and 3Mixtatin. - Outcomes: Success rates and adverse outcomes monitored over a specified duration.

Discussion

Pulpotomy is an essential method in clinical practice, if appropriately indicated through radiographical and clinical assessments. This procedure follows the propositions of minimal intervention by maintaining pulp vitality, reducing the need for pulpectomies and premature tooth extractions, ensuring the proper emergence of permanent teeth following the exfoliation of primary teeth, and enhancing the quality of life for children. Recent research indicates that pulpotomy remains a viable treatment option for vital pulp therapy in cases of carious exposure [4].

3Mixtatin was developed by combining the 3Mix antibiotic paste with simvastatin. Its success in pulpotomy procedures for primary teeth is likely attributed to its antibacterial, anti-inflammatory, and bio-inductive properties [20]. Specifically, the bio-inductive effect of simvastatin, a component of 3Mixtatin, stands out. Statins are utilized in regenerative dentistry because of their pleiotropic effects, which support bone formation by enhancing osteoblast activity and inhibiting osteoclast function [21,22]. Moreover, statins encourage osteoblastic differentiation from undifferentiated cells, increase the number of nerve cells, and induce vasculogenesis, potentially leading to regenerative endodontics [23].

Composition of 3Mixtatin

The composition of 3Mixtatin includes three antibiotics: ciprofloxacin (100 mg), metronidazole (100 mg), and cefixime (100 mg). Simvastatin, in a quantity of 2 mg, serves as the vehicle in this formulation.

Method of Preparation of 3Mixtatin

Each medication's enteric coating or capsule was taken out, and an analytical balance with a 0.1 mg precision was used to take precise measurements. Using a pestle and mortar, each medication was ground into a powder and kept in an airtight porcelain container to keep out light and moisture. The resulting powders were then mixed in equal proportions (1:1:1) as referenced by Jamali et al., Chak et al., Mushtaq et al, and Reddy et al. To this blend, 2 mg of simvastatin was added to create 3Mixtatin. This mixture was combined with normal saline on a clean glass slab using a spatula, forming a thick consistency.

Jamali et al. provide essential insights into the comparison of three pulpotomy materials - 3Mixtatin, formocresol, and MTA - which are used as pulpotomy agents in primary teeth. Their study revealed that 3Mixtatin had an overall success rate of 90.5%. The high success rate of 3Mixtatin in primary tooth pulpotomy is likely attributed to its antibacterial, anti-inflammatory, and bioinductive properties [16].

Chak et al. contributed valuable data to the expanding research on 3Mixtatin, which is formed by combining three antibacterial drugs with simvastatin. Their study demonstrated a 93.8% success rate in pulpotomized primary molars after a 12-month follow-up. The high success rate can be attributed to its antibacterial, sterilizing, anti-inflammatory, and bioinductive properties. Furthermore, its lack of cytotoxic effects on odontoblasts and the pro-inflammatory cytokine IL-6 may promote angiogenesis and reparative dentin formation, further enhancing its effectiveness [17].

Mushtaq et al. offer valuable insights into the treatment outcomes of 3Mixtatin and MTA as pulpotomy agents in primary teeth. According to their research, after a year, 3Mixtatin had a radiographic success rate of 91.3% and an overall clinical success rate of 95.7%. The success of 3Mixtatin is likely due to its ability to promote bone formation and stimulate osteoblastic activity. Its well-established pro-angiogenic properties may also aid in pulp regeneration in treated teeth when paired with statins suppression of pro-inflammatory cytokines [18].

Reddy et al. provided valuable insights into the comparison of three pulpotomy agents - formocresol, MTA, and 3Mixtatin - for use in primary teeth. Their study found that 3Mixtatin demonstrated success rates of 95% at six months and 85% at 12 months. Given its biocompatible and bioinductive properties, 3Mixtatin can be considered a promising additional option for pulpotomy in primary teeth [19].

On the other hand, MTA serves as a biologically active substrate that encourages the growth of human osteoblasts and facilitates the formation of thick dentine bridges [24]. Studies have shown that MTA achieves high radiographic success rates, ranging from 96% to 100%, in pulpotomized primary molars after a one-year follow-up [25,26]. MTA and its derivatives possess excellent biocompatibility and sealing abilities, creating an effective barrier against bacterial infiltration into the remaining pulp in the canals [9,27,28]. As a result, the development of new materials that are as effective as MTA is essential. The results of the review indicate that 3Mixtatin demonstrated slightly higher clinical and radiographic success rates compared to MTA. The studies consistently demonstrated high success rates for 3Mixtatin, positioning it as an effective alternative to traditional pulpotomy agents like MTA and formocresol, with some evidence suggesting slightly better radiographic outcomes for 3Mixtatin over shorter periods.

Out of all four studies, the clinical success rates for 3Mixtatin were consistently high across all included studies, demonstrating its potential as an effective alternative to traditional pulpotomy agents like MTA and formocresol. Radiographic success rates were similarly high, although specific rates for 3Mixtatin were often not detailed. The high success rates for 3Mixtatin can be attributed to its combined antibiotic and statin properties, which promote better healing and provide antibacterial effects. These properties likely contribute to its effectiveness in pulpotomy procedures, as noted in the main findings of the included studies. The results of this analysis provide credence to 3Mixtatin's prospective application as a practical material for pulpotomy treatments in pediatric dentistry. The consistently high success rates across various studies suggest that 3Mixtatin could be considered a reliable alternative to MTA, with the added benefits of its bio-inductive and antibacterial properties.

All trials, however, demonstrated a high risk of bias with regard to participant, staff, and outcome assessment blinding, as well as allocation concealment. While randomization was consistently applied across the studies, none explicitly detailed allocation concealment, introducing a risk of selection bias. Incomplete outcome data presented medium risk in two studies due to follow-up loss or exclusions, while the third study reported complete follow-up, minimizing bias in that aspect. Selective reporting was consistently low risk, and ethical approval was obtained in all studies, though limited design details resulted in a medium risk for two articles.

A key strength of this systematic review is the comprehensive search strategy employed across multiple databases, ensuring a thorough evaluation of existing literature. For the first time, data from clinical trials comparing 3Mixtatin and MTA as pulpotomy medications in primary teeth have been compiled in this systematic review. Additionally, the results were more reliable and less biased due to the independent screening and data extraction carried out by several reviewers. However, the review has some limitations. The limited number of included studies, along with variability in study designs, sample sizes, and follow-up periods, may restrict the generalization of the results. Furthermore, the possibility of bias in certain research could impact the general conclusions. Due to the small sample size of studies, only a systematic review was conducted, and a meta-analysis could not be performed. This restriction prevented the pooling of data and quantitative assessment of the overall effect, leading to the decision to focus solely on qualitative synthesis in the review.

To further validate the results of this analysis, future studies should strive to carry out carefully planned RCTs with bigger sample sizes and longer follow-up times. Comparative studies examining additional clinical outcomes, such as patient comfort and long-term tooth survival, would provide a more comprehensive understanding of the relative efficacy of MTA and 3Mixtatin. Additionally, exploring the cost-effectiveness of these materials could inform more practical decision-making in clinical settings.

## Conclusions

In conclusion, this systematic review highlights the high radiographic as well as clinical success rates of pulpotomy material, 3Mixtatin in primary teeth, comparable to or slightly better than MTA. The review underscores the importance of methodological rigour in future studies, particularly in aspects such as allocation concealment and blinding, to reduce potential biases. The findings support the clinical use of 3Mixtatin, given its effective antibacterial and bio-inductive properties, making it a promising material for pediatric pulpotomy procedures.
